# Single DNase or Proteinase Treatment Induces Change in Composition and Structural Integrity of Multispecies Oral Biofilms

**DOI:** 10.3390/antibiotics10040400

**Published:** 2021-04-07

**Authors:** Lamprini Karygianni, Pune N. Paqué, Thomas Attin, Thomas Thurnheer

**Affiliations:** Clinic of Conservative and Preventive Dentistry, Center of Dental Medicine, University of Zurich, Plattenstrasse 11, 8032 Zurich, Switzerland; Lamprini.Karygianni@zzm.uzh.ch (L.K.); thomas.attin@zzm.uzh.ch (T.A.); thomas.thurnheer@zzm.uzh.ch (T.T.)

**Keywords:** biofilm matrix, DNAse I, proteinase K, CLSM, antimicrobial, biofilm control

## Abstract

Biofilm virulence is mainly based on its bacterial cell surrounding biofilm matrix, which contains a scaffold of exopolysaccharides, carbohydrates, proteins, lipids, and nucleic acids. Targeting these nucleid acids or proteins could enable an efficient biofilm control. Therefore, the study aimed to test the effect of deoxyribonuclease I (DNase I) and proteinase K on oral biofilms. Six-species biofilms (*Streptococcus mutans*, *Streptococcus oralis*, *Actinomyces oris*, *Fusobacterium nucleatum*, *Veillonella dispar*, and *Candida albicans)* were exposed to DNase I (0.001 mg/mL, 0.002 mg/mL) or proteinase K (0.05 mg/mL, 0.1 mg/mL) for 1 h during biofilm formation. After 64 h, biofilms were harvested, quantified by culture analysis and visualized by image analysis using CLSM (confocal laser scanning microscopy). Statistical analysis was performed by ANOVA, followed by the Tukey test at a 5% significance level. The biofilm treatment with proteinase K induced a significant increase of Logs_10_ counts in *S. mutans* and a decrease in *C. albicans*, while biofilm thickness was reduced from 28.5 μm (control) to 9.07 μm (0.05 mg/mL) and 7.4 μm (0.1 mg/mL). Treatment with DNase I had no effect on the total bacterial growth within the biofilm. Targeting proteins of biofilms by proteinase K are promising adjunctive tool for biofilm control.

## 1. Introduction

Prophylaxis and prevention strategies, in contrast to care intervention are nowadays gaining weight to maintain oral health and combat incipient oral diseases [1,2,3]. The oral microbiome, which consists of a high diversity of oral microorganisms [4,5], plays a pivotal role in the development of oral diseases, namely caries and periodontitis [6,7,8,9]. Both diseases are caused by oral biofilms, which develop and mature on biotic and abiotic surfaces. Oral biofilms are best described as “aggregates of microorganisms, living in a self-produced matrix of hydrated extracellular polymeric substances (EPS) that form their immediate environment” [10]. The microbial cell surrounding EPS contributes highly to pathogenic biofilm properties and the development of oral infections [11]. It is mainly composed of polysaccharides, proteins, lipids and DNA [10]. Major EPS functions include bacterial adhesion and cohesion, as well as scaffolding processes. Furthermore, mechanical stability and protection against dispersal, antimicrobial agents and desiccation is provided. The complex EPS composition and functional diversity is termed the biofilm matrixome [12]. Its unique impact on biofilm virulence is increasingly studied and deepened in recent investigations. Hence, antibiofilm strategies to combat oral diseases are not only focusing on the sole eradication of microorganisms anymore. Deficient response to conventional treatments and increasing drug tolerances through biofilm properties indicate novel or multi-targeted antibiofilm approaches [13,14]. Targeting EPS functional and structural integrity, for example, might influence integral biofilm composition and organization, based on its scaffolding and stability qualities. Extracellular DNA (eDNA) and matrix proteins are essential contributor to biofilm stability [15,16,17]. DNA release to the extracellular environment is based on bacterial autolysis of streptococcal cells, such as *Streptococcus mutans* [18], or during hydrogen peroxide-based microbial competition strategies without cell lysis [19]. In turn, DNase presence in *S. mutans* biofilm media enriched with sucrose and starch can result in biomass decrease [20]. Matrix proteins are, next to other functions, involved in the 3D biofilm architecture [21]. Their inhibition by special antibodies or proteinases induces changes in bacterial co-binding within biofilms and matrix degradation with increase of nucleic acid release [22,23]. Novel multi-targeted antibiofilm approaches, applying these or other targets, could provide more insights into effective biofilm treatment strategies [24]. 

The Zurich biofilm model was established more than a decade ago and was designed as a fully defined, in vitro batch model system used as a supragingival model consisting of six oral microorganisms characteristic for supragingival plaque. Previous studies showed that this biofilm model is a most reliable tool e.g., to predict the in vivo efficacy of antimicrobial or antibiofilm agents [25]. In an earlier study we showed the impact of the combination of DNase I and proteinase K on our supragingival biofilm model [24]. Therefore, the aim of this study was the analysis of multispecies biofilm composition and structural integrity after single DNase I or proteinase K treatment using different enzyme concentrations. Change in biofilm architecture was visualized after enzymatic treatment using fluorescence staining and confocal laser scanning microscopy (CLSM). Effects on bacterial counts were determined by colony forming units (CFU). The null hypothesis of the study implies that none of the enzymatic treatments using DNase I or proteinase K can induce changes in multispecies biofilm composition or structural integrity. 

## 2. Results

### 2.1. Culture Analyses

**Figure 1:**
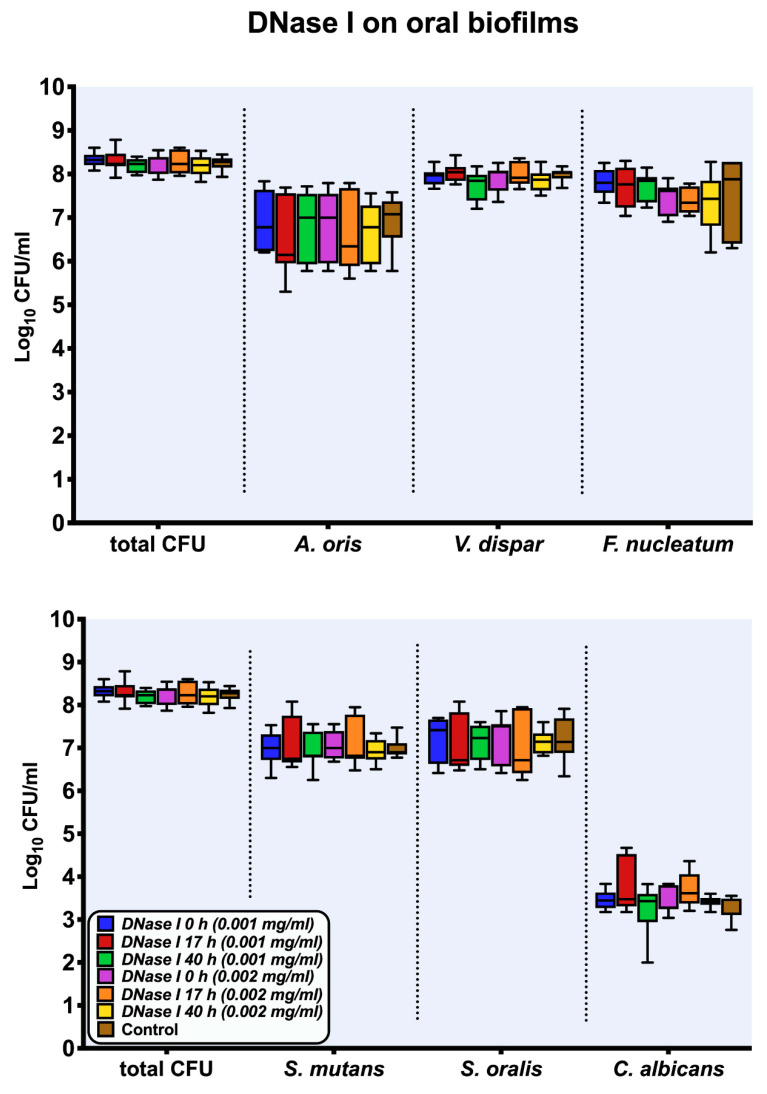
Boxplots with median and interquartile ranges show the results of the culture analyses (colony forming units (CFUs)) of the multispecies biofilms after one-hour exposure to DNase I (0.001 mg/mL, 0.002 mg/mL) at intervals of 0 h, 17 h, and 40 h, respectively. The untreated biofilms are presented as negative controls and CFUs are shown on a log_10_ scale per milliliter (Log_10_/mL).

Figure 1 demonstrates the microbial growth rates log_10_ counts of six-species supragingival biofilms incubated over 64 h in vitro after one-hour exposure to DNase I (0.001 mg/mL, 0.002 mg/mL) at specific intervals (0 h, 17 h, 40 h). No significant discrepancies could be detected regarding the effects of the low (0.001 mg/mL) versus high (0.002 mg/mL) tested DNase I concentrations. Incubation of the biofilms with two different concentrations of DNase I (0.001 mg/mL, 0.002 mg/mL) had also no effect on the total bacterial counts. No significant differences were detected between different concentrations of DNase I and proteinase K independent of the time of their addition (0 h, 17 h, 40 h) into the biofilm mass.

**Figure 2:**
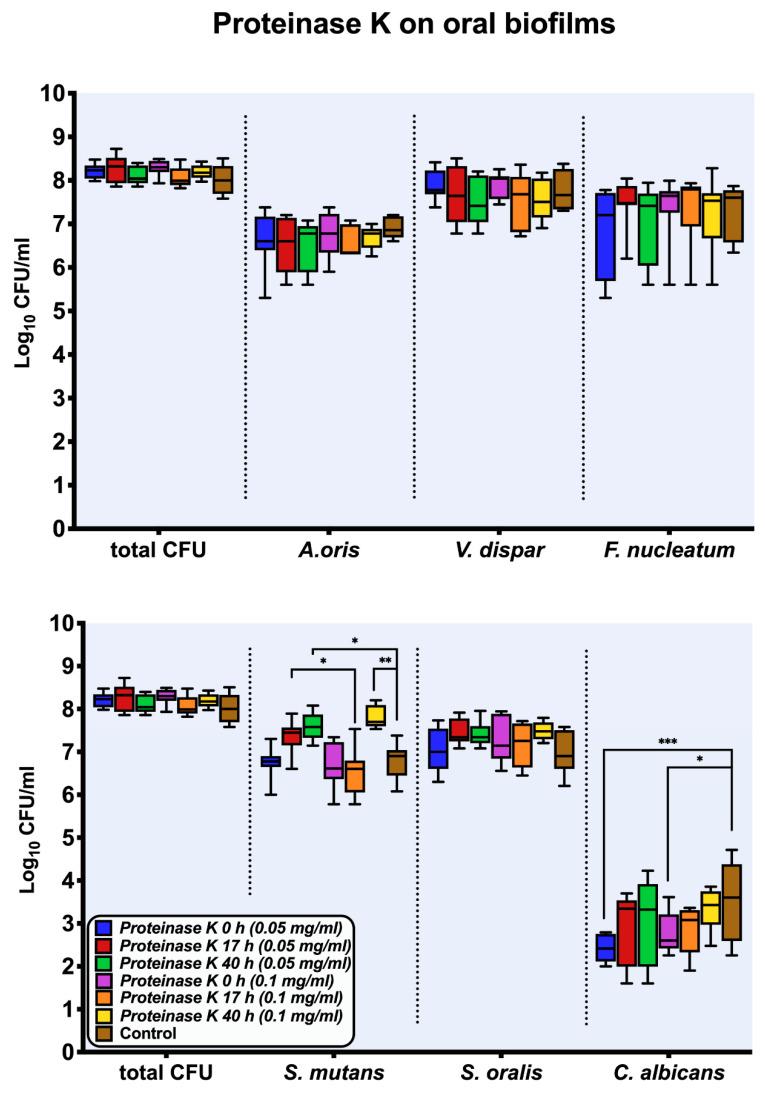
Boxplots with median and interquartile ranges show the results of the culture analyses (CFUs) of the multispecies biofilms after one-hour exposure to proteinase K (0.05 mg/mL, 0.1 mg/mL) at intervals of 0 h, 17 h, and 40 h, respectively. The untreated biofilms are presented as negative controls and CFUs are shown on a log_10_ scale per milliliter (Log_10_/mL). Asterisks show statistically significant differences (* *p* < 0.033, ** *p* < 0.002, *** *p* < 0.0002).

Figure 2 shows the log_10_ counts of in vitro six-species supragingival biofilms grown for 64 h after one-hour exposure to two different concentrations of proteinase K (0.05 mg/mL, 0.1 mg/mL) at specific intervals (0 h, 17 h, 40 h). After treatment with proteinase K, all treated biofilms demonstrated log_10_ counts similar to that of the negative control. However, the log_10_ counts of two individual microbial species (*Streptococcus mutans*, *Candida albicans*) changed significantly after biofilm treatment with proteinase K at specific intervals (0 h, 17 h, 40 h). Specifically, biofilm treatment at 40 h with 0.1 mg/mL proteinase K (mean, 7.801 ± 0.25 log_10_ CFU; *p* = 0.0013) and at 40 h with 0.05 mg/mL proteinase K (mean, 7.605 ± 0.29 log_10_ CFU; *p* = 0.0213) induced an increase in *S. mutans* CFU counts when compared to the untreated control (mean, 6.793 ± 0.43 log_10_ CFU). *C. albicans* CFU counts presented a reduction after biofilm treatment at 0 h with 0.05 mg/mL proteinase K (mean, 2.436 ± 0.32 log_10_ CFU; *p* = 0.0002) and at 0 h with 0.1 mg/mL proteinase K (mean, 2.792 ± 0.44 log_10_ CFU; *p* = 0.0375) when compared to the untreated control (mean, 3.558 ± 0.89 log_10_ CFU). 

### 2.2. Image Analyses

**Figure 3:**
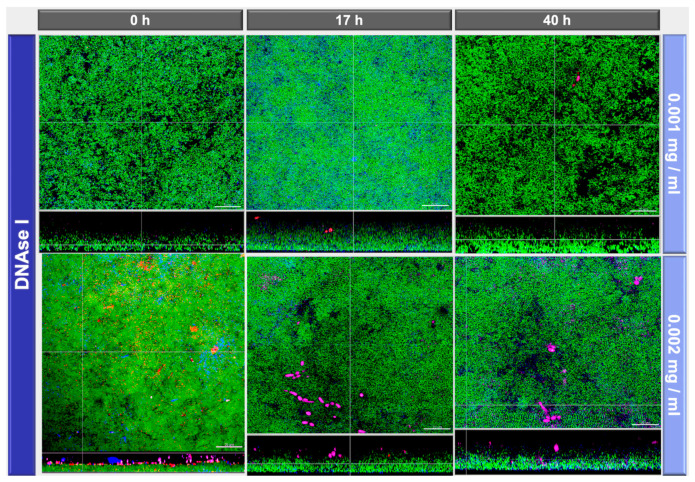
CLSM (confocal laser scanning microscopy) images show the multispecies biofilms after deoxyribonuclease I (DNase I) exposure at two concentrations (0.001 mg/mL, 0.002 mg/mL) and intervals (0 h, 17 h, 40 h). Bacteria are stained green (Yo Pro 1/Sytox Green DNA-staining), extracellular polymeric substances (EPS) blue (calcofluor), extracellular DNA appear red (anti-DNA antibodies and streptavidin, Cy3), and extracellular proteins magenta (SYPRO Ruby). The cross section shows the base of the biofilm base, which is directed towards the top view with a scale of 20 µm.

**Figure 4:**
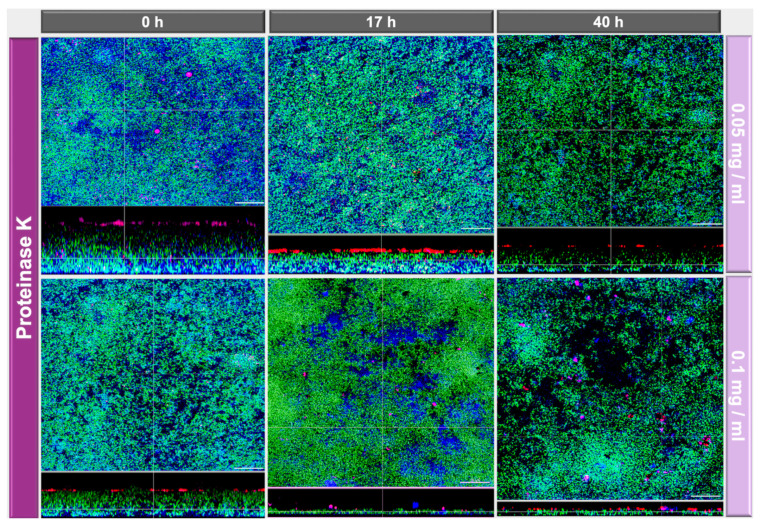
CLSM images show the multispecies biofilms after proteinase K exposure are two concentrations (0.05 mg/mL, 0.1 mg/mL) and intervals (0 h, 17 h, 40 h). Bacteria are stained green (Yo Pro 1/Sytox Green DNA-staining), extracellular polymeric substances (EPS) blue (calcofluor), extracellular DNA appear red (anti-DNA antibodies and streptavidin, Cy3), and extracellular proteins magenta (SYPRO Ruby). The cross section shows the base of the biofilm base, which is directed towards the top view with a scale of 20 µm.

**Figure 5:**
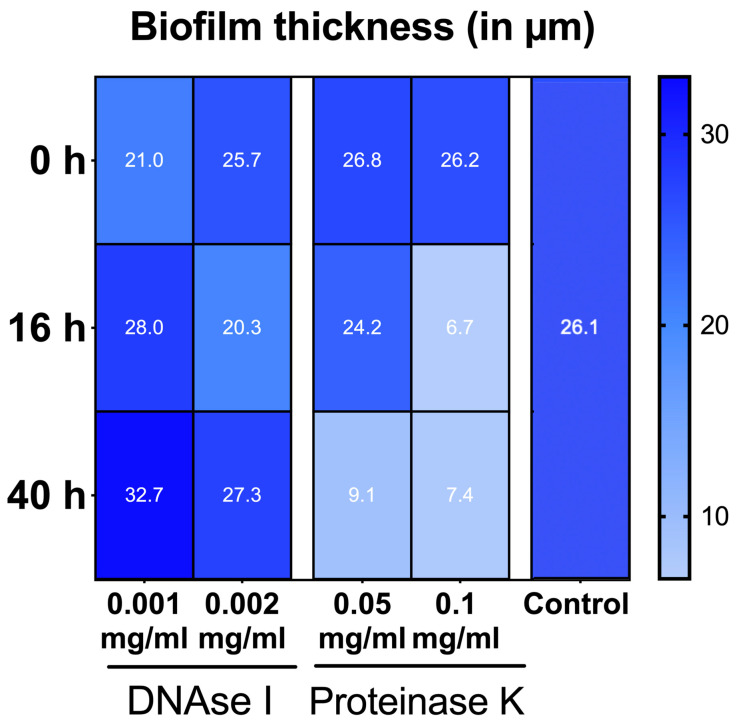
Heat maps of the mean thickness of multispecies biofilms after one-hour exposure to DNase I (0.001 mg/mL, 0.002 mg/mL) and proteinase K (0.05 mg/mL, 0.1 mg/mL) at intervals of 0 h, 17 h, 40 h. After visualization of the treated biofilms with immunofluorescence (IF) and confocal laser scanning microscopy (CLSM) using anti-DNA antibodies, streptavidin (Cy3), calcofluor, SYPRO Ruby and YoPro-1/Sytox, the quantitative analysis of the CLSM images was conducted with IMARIS.

The effects of DNase I and proteinase K treatment of the in vitro biofilms are shown in representative CLSM images in Figure 3 and Figure 4, respectively. In the upper row the effects with low concentrated DNase I and proteinase K (0.001 mg/mL and 0.05 mg/mL, respectively) are shown, the lower row shows the effects with high concentrated DNase I and proteinase K (0.002 mg/mL + 0.1 mg/mL, respectively). From left to right enzymatic treatment after 0 h, 17 h, and 40 h is shown. Biofilm organisms are stained green, extracellular polysaccharides appear blue, eDNA is stained red and extracellular proteins magenta. The effect of 0.001 mg/mL DNase treatment is not so prominent whereas 0.002 mg/mL DNase treatment resulted in less dense biofilms and loss of eDNA (Figure 3). Interestingly, DNase treatment resulted also in loss of extracellular polysaccharides (Figure 3). On the other hand, proteinase K treatment had no visible effect regarding extracellular polysaccharides but it resulted also in less dense biofilms and additionally had a big impact on biofilm thickness, the greatest effect with 0.1 mg/mL proteinase and treatment being observed after 40 h (Figure 4). This reduction of the biofilm thickness as revealed by the CLSM images from 26.2 ± 1.8 µm (0 h) to 6.7 ± 1.8 µm (17 h) and 7.4 ± 0.5 µm (40 h) after treatment with 0.1 mg/mL proteinase is illustrated in Figure 5.

## 3. Discussion

The present study focused on the effect of different DNase I and proteinase K concentrations on oral multispecies biofilms, by single intervallic use during biofilm formation. For the first time, both enzymes were investigated separately on the same multispecies biofilms, which were quantified with CFU counts and visualized using multi-targeted fluorescent markers to detect the biofilm mass, matrix, and extracellular DNA proteins. Bacterial growth after the enzymatic treatment with DNase I or proteinase K was quantified by the determination of CFU on agar media, which not only constitutes a representative cultural approach for assessing the antibiofilm effectiveness of the enzymatic therapy protocols, but is also the most widely used technique to estimate biofilm cell viability. Based on the universal dilution series approach used to quantify cells, this technique is available in every microbiological laboratory.

Interestingly, both DNase I concentrations (0.001 mg/mL or 0.002 mg/mL) did not cause measurable bacterial shifts using culture analyses, which is slightly different from the results of a previous study where a reduction of CFU after DNase I treatment could be observed for *A. oris*, *C. albicans*, *F. nucleatum*, *S. mutans*, and *S. oralis* [24]. In that experiment, however, the biofilms were exposed to the enzymes for the entire duration of the experiment, whereas in the present study the biofilms were only exposed to the enzymes at specific times. The visualization of DNase-treated biofilm, however, demonstrated less dense biofilms after 0.002 mg/mL DNase I treatment, as well as reduced eDNA and EPS structures. Proteinase K caused an increase of *S. mutans* counts and a decrease of *C. albicans* in the multispecies biofilm. The biofilm structure was also affected by proteinase K treatment, resulting in less dense biofilms. Additionally, a dose-dependent effect on biofilm thickness was observed, resulting in 7.43 µm after 40 h biofilm formation and intervallic application of 0.1 mg/mL proteinase K, compared to the control biofilm thickness of 28.54 µm. The low-level proteinase treatment (0.05 mg/mL) caused less prominent reductions in biofilm thickness after 17 h (24.18 µm) and more pronounced effects, if applied after 40 h (9.07 µm), respectively.

The change in microbial composition of the multispecies biofilms after DNase I and proteinase K treatment was assessed using culture analyses. The quantification of species-specific shifts was thereby facilitated using selective agars. Image analysis using the CLSM after fluorescence staining of biofilm cells (stained with Yo Pro 1/Sytox green) and biofilm matrix components (EPS stained with Calcofluor, eDNA with Cy3-streptavidin labelled anti-DNA-antibody, and extracellular proteins with Sypro^TM^ Ruby) enabled the visualization of the hydrated three-dimensional biofilms and its structural integrity after enzymatic exposure. 

The impact of eDNA for biofilm formation was described by Whitchurch and co-workers, who applied DNase I during biofilm formation using a *Pseudomonas aeruginosa* flow-chamber system [26]. DNase I application during biofilm formation prevented the development of biofilms. Yet, DNase I exposure on established biofilms exhibited dissolving properties in a biofilm-age depending manner. The authors concluded that prophylactic DNase I measures might prevent chronic *P. aeruginosa* biofilm infections in cystic fibrosis risk patients. 

Yu and coworkers compared DNase application during biofilm formation and as treatment on matured endodontic *E. faecalis* biofilms after 2 days [27]. The bacterial counts, eDNA level, biofilm formation, and EPS volume were lower when DNase was applied during biofilm development compared to a single DNase application to matured biofilms. Similar results were observed in an endodontic study investigating DNase effects on *E. faecalis* cell adhesion during initial biofilm formation and dispersal. The microscopic analyses using eDNA stains revealed that DNase was able to remove adhering eDNA after 1 h, but not after 24 h of biofilm formation [28]. In our study, DNase was applied during biofilm formation, however, only at three timepoints for 1 h each. The intervallic exposure resulted in changes of the biofilm microstructure, however not in significant bacterial shifts compared to control biofilms. We detected a dose-dependent effect on biofilm density and a loss of eDNA, when 0.002 mg/ml DNase were applied compared to the low dose of 0.001 mg/mL DNase. This dose-dependent reduction is in line with a study, investigating *Streptococcus pneumoniae* clinical isolates after a staining of the matrix with the eDNA stain PicoGreen [29]. The pneumococcal biofilm biomass was significantly reduced after DNase treatment in a dose responsive manner. The authors also indicate that DNase treatment did not affect the maximum biofilm thickness and suggest that EPS matrix in biofilm towers might consist of non-DNA components. Distinct biofilm towers are not observed in our biofilm model; however, this might explain sparse DNase effects on biofilm thickness in other studies [27,29,30]. 

These results may also indicate, that plain DNase application on matured biofilms might not suffice to inhibit multispecies biofilm formation. Yet, a recently published endodontic study investigated DNase effects alone or in combination with an antimicrobial peptide Melittin on *E. faecalis* 7 days of biofilm development [30]. Interestingly, plain DNase application on the matured biofilm resulted in significantly lower bacterial counts and eDNA levels compared to the control biofilms. Though, biofilm formation was only significantly reduced, if a combined treatment of Melittin and DNase was applied. The authors suggested that DNase application might serve as pretreatment for biofilms and be used in combination with Melittin or other antimicrobial adjuncts to improve the antibiofilm efficacy against *E. faecalis* biofilms. 

The dynamics of biofilm killing and disruption were investigated by Niazi et al. [31] using 1% trypsin and 1% proteinase K on an endodontic multispecies biofilm model. The CLSM analyses of biofilms with live/dead staining resulted in effective bacteria killing and culture analyses revealed significant reductions of viable counts compared to the negative control. A follow-up study of the same group investigated the synergistic effect of 2% chlorhexidine alone or combined with 1% trypsin or 1% proteinase K during different irrigation protocols [32]. The root canal disinfection was significantly improved after the implementation of the proteolytic enzymes. These results are in line with our study, as proteinase k application reduced the biofilm thickness from 28.54 µm (control) to 9.07 µm (0.05 µg/mL) and 7.43 µm (0.1 mg/mL), if applied after 40 h of biofilm growth, respectively. Additionally, the whole biofilm structure was changed, being less dense after proteinase k treatment compared to the control. Lim and coworkers [33] targeted eDNA, proteins, and cellulose of *Escherichia coli* biofilms with DNase I, proteinase K and cellulase during development or as application on preformed biofilms. Single proteinase K or cellulase during biofilm development resulted in a reduction of the bacterial counts, while combined application of either proteinase K, DNase I, and cellulase followed by NaOCl showed significant reductions, with proteinase K revealing overall the most effective inhibition of biofilm formation or degradation of preformed biofilms. In our study, the single intervallic application of proteinase K, showed that proteinase K induced also a change in the bacterial biofilm composition. The decrease of 1.5 Logs_10_ in *C. albicans* was concomitant with an increase of 1.5 Logs_10_ in *S. mutans*. Although proteinase K does not discriminate between bacterial proteins and other proteins, its effects were evaluated for both microbial growth and matrix within oral biofilms. In our study, treatment with proteinase K during biofilm formation for 64 h facilitated growth of *S. mutans* (Figure 2). This may be related to the fact that amino acids may serve as nutrients for streptococci, namely *S. mutans*, in addition to neutralizing the acidity deriving from the metabolism of carbohydrates [34,35]. Furthermore, the proteinase K-mediated deactivation of quorum-sensing- or competence-stimulating peptides affects interspecies competition and thus, the prevalence of specific bacterial species such as *S. mutans* within biofilms. The detailed and underlying cause of this composition change and measured biofilm thicknesses is not clarified yet. 

The combination of proteinase K and DNase I were examined in a previous study, resulting in a synergistic effect of both enzymes and affecting the structural integrity of the biofilms [24]. In contrast, Ali Mohammed et al. [23] applied DNase I and proteinase K on *Fusobacterium nucleatum* and *Porphyromonas gingivalis* during biofilm formation and on mature biofilms using a dynamic and static biofilm model. Interestingly, both enzymes had only little effect on the biofilm matrix. The effects of single DNase I and proteinase K treatment on *Lactobacillus plantarum* biofilms were investigated by George and Halami [36] using fluorescence microscopy and viable cell counts. A notable decrease in cell density was observed in the enzymatic treated biofilms, while the untreated biofilms revealed dense live and dead cells. In our study, the single application of DNase I and proteinase K led to a loss of biofilm density and proteinase K led to a significant decrease in biofilm thickness and can therefore reduce matrix integrity. Additionally, proteinase K treatment independent of the applied concentration affected the prevalence of specific bacterial species within the biofilm such as *S. mutans* and *C. albicans* and thus, the ecological balance among six-species oral biofilms. In turn, treatment with DNase I had no effect on the bacterial growth within the oral biofilms. 

The anti-biofilm strategy to use enzymes that can dissolve the biofilm matrix (e.g., DNase and proteinase) seems to be a promising tool in order to increase biofilm susceptibility to antibiotics. The role of matrix proteins in oral biofilms has to be further investigated, especially with focus on further synergistic effects after combination with other irrigants or antiseptics.

## 4. Materials and Methods 

### 4.1. In Vitro Biofilm Experiments

The multispecies biofilms were produced as previously described [24,37,38]. Briefly, *Actinomyces oris* OMZ 745, *Candida albicans* OMZ 110, *Fusobacterium nucleatum* KP-F2 (OMZ 596), *Streptococcus oralis* SK 248 (OMZ 607), *Streptococcus mutans* UA159 (OMZ 918), and *Veillonella dispar* ATCC 17748^T^ (OMZ 493) were transferred from Columbia blood agar (CBA) into modified fluid universal medium (mFUM) [39]. The bacterial suspension was incubated at 37 °C under anaerobic conditions for 16 h and then again for 8 h. The inoculum was produced using equal volumes of all precultures after adjusting the optical density to OD_550_ = 1.0. 

Prior to biofilm formation, sintered hydroxyapatite disks (HA; Ø 9 mm, Clarkson Chromatography Products, Inc., South Williams-port, PA 17702, USA) were covered with whole un-stimulated and pooled saliva (further termed saliva) for 4 h to allow pellicle formation. The detailed procedure for the processing of saliva was described by Guggenheim et al. [39]. Biofilm formation was initiated using 1.6 mL of growth medium (70% saliva, 30% mFUM supplemented with Sørensen’s buffer with pH 7.2) and 200 µL of the inoculum to cover the pellicle-coated disks. The bacterial suspension was incubated anaerobically for 64 h. At specific intervals (0 h, 16 h, 40 h) biofilms were exposed to two different concentrations of DNase I (0.001 mg/mL or 0.002 mg/mL) and proteinase K (0.05 mg/mL or 0.10 mg/mL) for 1h. The timing of exposure and incubation time as well as enzyme concentrations were optimized in preliminary experiments (data not shown). At specific time points (24 h, 44 h, and 48 h) and after exposure to enzymes (0 h, 16 h, or 40 h), disks were dipped in saline (3x) to remove remaining enzymes and loose microorganisms from the disks. Fresh medium was used after 17 h and after 40 h of biofilm growth. After biofilm formation (64 h), disks were used for culture analyses and confocal laser scanning microscopy (CLSM). Nine biofilms were quantified with culture analyses per experimental group, resulting in a total of 126 biofilms (DNase I and proteinase K with two concentrations at three different timepoints, and the untreated control).

The culture analyses were performed after vortexing the biofilm-covered disks in 1 mL of 0.9% NaCl (1 min). The generated biofilm suspension was further sonicated (30 W for 5 s, Sonifier B-12, Branson Ultrasonic, Urdorf, Switzerland) to facilitate bacterial dispersal. A dilution in saline was performed serially and aliquots of 50 µL were plated on human blood (5%) supplemented CBA base (Oxoid Ltd., Basingstoke, UK). Species-specific bacterial counts were enabled using selective agars as described earlier [39,40]. In brief, total bacterial counts, *A. oris* and *V. dispar* were obtained using CBA plates, whereas *S. mutans* and *S. oralis* were detected on Mitis Salivarius Agar (Difco Laboratories, Inc., Detroit, MI, USA) supplemented with 0.001% (*w*/*v*) sodium tellurite. Fastidious Anaerobe Agar (Chemie Brunschwig, Basel, Switzerland) was used to enumerate *F. nucleatum* and BIGGY Agar (BBL, Becton Dickinson, Allschwil, Switzerland) for *C. albicans*. Colony morphology enabled the identification of all species after incubation of the agar plates incubated at 37 °C for up to 72 h.

### 4.2. Biofilm Staining and Confocal Laser Scanning Microscopy (CLSM)

The biofilm-covered disks (at least two per group) were fixed and washed before staining and confocal laser scanning microscopy. For the fixation, 4% paraformaldehyde and RNase inhibitor (RNAi) were applied on the biofilms for 2 h at 4–8 °C. The washing step was performed using 500 μL 0.9% NaCl and RNase Inhibitor, followed by blotting the biofilms gently on a paper towel. Gram-positive bacteria were permeabilized in a 1 mg/mL lysozyme solution (Sigma, Buchs, Switzerland; 70V000 U/mL) in 0.1 M Tris-HCl, pH 7.5, 5 mM EDTA (8 min, room temperature) and rinsed with 0.9% NaC. 

The mixture of 3 μM YoPro 1 iodide (Invitrogen, Basel, Switzerland) and 15 μM Sytox green (Invitrogen) in nanopure water (30 min in the dark) was used to stain total DNA. The biofilm matrix was then stained with Calcofluor (Sigma; 10 µg/mL solution in 10 mM sodium phosphate, pH 7.5) by incubation the biofilm disks for 30 min at room temperature in the dark. The Cy3-streptavidin labelled anti-DNA-antibody (Sigma-Aldrich, Buchs, Switzerland) was applied for the staining of the extracellular DNA (eDNA), and Sypro Ruby for the extracellular proteins, each following the manufacturer’s protocols. The biofilm samples were fixed on upside down chamber slides using 100 μL of Mowiol [39].

The biofilm samples were visualized with a Leica TCS SP5 microscope (Leica Microsystems, Wetzlar, Germany) from the Centre for Microscopy and Image Analysis of the University of Zurich as previously described [41]. A UV laser (405 nm excitation), an Argon laser (488 nm), a DPSS diode laser (561 nm), and a Helium-Neon laser (633 nm) were applied and adjusted at 430–470 nm (Calcofluor), at 500–540 nm (Yo Pro 1/Sytox green), at 570–600 nm (Cy3), and at 660–710 nm (Sypro Ruby). Sequential scans (1 μm thickness) were selected and generated images of the biofilms were processed using Imaris 8.3 (Bitplane, Zurich, Switzerland). 

### 4.3. Statistics

The biofilm experiments were performed in triplicate biofilm cultures resulting in nine individual biofilm cultures per experimental group. For the statistical analysis, a two-way analysis of variance (ANOVA) was applied to assess differences in bacterial counts between the control group (untreated biofilm) and the enzymatically treated biofilms. The Tukey’s multiple comparisons test was applied for correction and all missing values were imputed as lowest detection limit value of the assay to allow for logarithmic transformation. All statistical analyses were performed with GraphPad Prism (version 7) to compare the species’ total cell counts within the different biofilm groups. The significance level was set at *p* < 0.05. 

## 5. Conclusions

Single DNase I and proteinase K treatments reduce biofilm density and thickness. Proteinase K can affect the microbial composition and presumably ecological balance among six-species oral biofilms.
